# What young people say about impulsivity in the short-term build up to self-harm: A qualitative study using card-sort tasks

**DOI:** 10.1371/journal.pone.0244319

**Published:** 2020-12-21

**Authors:** Joanna Lockwood, Ellen Townsend, Heather Allen, David Daley, Kapil Sayal

**Affiliations:** 1 Division of Psychiatry & Applied Psychology, University of Nottingham, Nottingham, United Kingdom; 2 Self-Harm Research Group, School of Psychology, University of Nottingham, Nottingham, United Kingdom; 3 NIHR MindTech MedTech Co-operative, Institute of Mental Health, School of Medicine, University of Nottingham, Nottingham, United Kingdom; Institute of Mental Health, SINGAPORE

## Abstract

Youth who self-harm report high levels of trait impulsivity and identify impulsive behaviour as a proximal factor directly preceding a self-harm act. Yet, impulsivity is a multidimensional construct and distinct impulsivity-related facets relate differentially to self-harm outcomes. Studies have yet to examine if and how a multidimensional account of impulsivity is meaningful to individual experiences and understandings of self-harm in youth. We explored the salience and context of multidimensional impulsivity within narratives of self-harm, and specifically in relation to the short-term build-up to a self-harm episode. Fifteen community-based adolescents (aged 16–22 years) attending Further Education (FE) colleges in the UK took part in individual face-to-face sessions (involving exploratory card-sort tasks and semi-structured interviews) which explored factors relating to self-harm, impulsivity and the broader emotional, developmental and cognitive context. Session data were analysed thematically. Two overarching themes, and associated subthemes, were identified: ‘How I respond to strong negative emotions’; and ‘Impulse versus deliberation- How much I think through what I’m doing before I do it’. Self-harm was typically a quick, impulsive act in the context of overwhelming emotion, underpinned by cognitive processing deficits. The dynamic tension between emotion-based impulsivity and controlled deliberation was articulated in the immediate moments before self-harm. However, impulsive responses were perceived as modifiable. Where self-harm patterns were established, these related to habitual behaviour and quick go-to responses. Young people identified with a multidimensional conception of impulsivity and described the impulsive context of a self-harm act as dynamic, contextual, and developmentally charged. Findings have implications for youth-focused work. Card-task frameworks are recommended to scaffold and facilitate discussion with young people, particularly where topics are sensitive, complex and multifactorial.

## Introduction

Self-harm is a common and often repeated behaviour affecting young people, with rates on the increase in the UK, notably among females aged 16–24 years [1]. A rich body of qualitative literature has explored the ways in which self-harm is understood and experienced by adolescents in community-based settings [2–5]. Yet, understanding of the influence of complex constructs–such as impulsivity–on self-harm processes in young people is drawn largely from quantitative studies, which have sought to establish psychological mechanisms as population-based correlates of and risk factors for self-harm. As such, there is still much to uncover about how such psychological processes are actually understood or experienced within individual self-harm narratives. Here self-harm is defined according to NICE (National Institute for Health and Care Excellence) guidelines (NICE, 2014) as self-injury or self-poisoning regardless of the intention and motivation behind the act, which recognises the dimensional structure of self-harmful behaviour [6].

Impulsivity is a multifaceted construct comprising a dispositional tendency towards rash action, alongside (and distinct from) performance-based behavioural indicators of impulsive action or impulsive choice [7]. The UPPS-P model of impulsive behaviour [8, 9] distinguishes five separate, though related, personality-based pathways to impulsive behaviour, allowing greater precision in empirical tests of associations between impulsivity facets and behavioural outcomes. The model suggests that emotion-based impulsivity, in which rash action occurs in the presence of heightened negative emotion (Negative Urgency) or positive emotion (Positive Urgency), can be differentiated from facets of impulsivity which relate to deficits in conscientiousness, where impulsive action is a result of poor deliberation (lack of Premeditation), or difficulties maintaining a course of action when tasks are found boring or challenging (lack of Perseverance). In addition, impulsive behaviour may derive from a drive for novelty and experience seeking (Sensation Seeking). Broadly self-harm is best characterised by Negative Urgency [10, 11]. Recent lab-based work suggests this process may relate to difficulties in inhibiting negative emotional responses once urges have passed a threshold of intensity [12].

Though not explicitly focused on impulsivity, qualitative findings have nonetheless pinpointed impulsive processes within adolescent self-harm. Adler & Adler [4] conducted eighty in-depth interviews with adolescents and adults documenting the sociological and psychological ways in which participants described the practice and process of self-harm. Participants identified difficulties controlling the impulse to self-harm and described feeling at the whim of the urge–an irresistible-impulse model of self-harm. Hill and Dallos [2] interviewed six young people aged 13–18 to explore how they saw self-harm as fitting within the broad narrative of their lives. Self-harm emerged via individual accounts as a quick response to negative moods, a “short-way round of feeling better”, in which, crucially there was no necessity to “think everything through” [4, p.467). Young people have consistently articulated the emotional dynamics that underlie pathways to self-harm, suggesting that self-harm provides a temporary immediate resolution to heightened emotional state [3, 13]. For some, however, the processes driving self-harm appear to be deliberative rather than impulsive. Adler and Adler [4] describe accounts of planned behaviour in which the decision to self-harm follows a process of thoughtful consideration in which urge can be consciously delayed, stored for later retrieval. Paradoxically then, a quick response to an urge, could be a product of earlier thought-through deliberation.

Conceptions of irresistible impulse-driven behaviour are consistent with performance-based accounts of impulsive action or an inability to refrain from behaviour in the moment [7]. As such, these accounts may emphasise a perceived proximal relevance of impulsivity, and inadequate cognitive restraint, in the immediate moments prior to a self-harm act, rather than a general (distal) contribution conferred by an impulsive disposition [14]. Theorists have debated the proximal versus distal influence of impulsivity on self-harm and suicidality, implicating impulsivity as a proximal volitional moderator involved in the translation of thought to act [15], or a distal risk factor which relates to self-harm and suicidality via the increased painful or provocative experiences that being prone to impulsiveness creates [16]. In their cognitive model of suicidal behaviour, [17] Wenzel and Beck suggest that dispositional vulnerability factors (including impulsivity) confer non-specific risk distally (e.g. by increasing life stress), but also serve to increase immediate proximal risk by distorting cognitive processes at the acute moment of distress. Such distortions include attentional fixation (i.e. narrowed focus on a course of action). A suicidal act follows when the culmination of suicide-relevant cognitive processes passes a threshold of tolerance. Impulsivity may contribute to the faster activation of this process. Relatedly, Townsend and colleagues systematically investigated the dynamic interplay between distal and proximal factors that lead up to and follow an act of self-harm in young people using the novel Card Sort Task for Self-harm [CaTS;18]. Participants selected from a bank of cards describing thoughts, feelings, event and behaviours, choosing those relevant to an act of self-harm and placing these in order along a delineated timeline from 6 months until the point of self-harm. Using lag sequential analysis, they found that among multiple potential risk factors, broadly specified impulsivity (“I did it on impulse without thinking”) was the only salient immediate precursor to behavioural enaction of self-harm. Qualitative examinations may contribute to these debates by drawing out individual interpretations of the relationship between self-harm and facets of impulsivity (and other self-regulatory or cognitive processes) at a dispositional (trait) level and in relation to the build-up to a self-harm act.

### The current study

This study sought to explore young people’s understanding and experience of self-harm in the context of impulsivity-related behaviour. As part of this, we sought to unpick the salience of impulsivity and the wider emotional/cognitive context as a distal/proximal influence in the build-up to a self-harm act, through dialogue with young people during qualitative interviews. Crucial to the approach was the starting point that young people would have sufficient understanding–or conscious awareness–of the internal processes involved in their thinking, feeling and action, such that they could identify and describe how such processes related to self-harm [See 19]. Evidence suggests participants can recall and articulate insight into their own behaviours in research settings [e.g. 3]. Nonetheless, methods of data collection which facilitate understanding may be useful when discussing a sensitive, complex and multifaceted phenomenon such as self-harm, and especially in youth for whom the processing and describing of their own thinking, emotions and responses may still be developing [20]. We included two card-sorting tasks within interview sessions as a means of scaffolding understanding and providing a structured springboard for dialogue. The methodology aimed to facilitate conversation, scaffold psychological understanding, and provide access to adolescents’ experiences and understanding of impulsivity, and the emotional and cognitive context of self-harm.

## Methods

Participants were recruited following participation in a previous online survey of n = 374 young people (76.7% white and 59.1% female) aged 16–22 years (mean age = 17.18) across four FE colleges in the East Midlands of England. The survey examined psychological processes, including impulsivity, anxiety and depression, emotion regulation and distress tolerance alongside self-harm thoughts and acts. Prevalence of lifetime self-harm in the survey was 35% which is comparable to rates reported in mid-to-late adolescent samples in community-based studies [21, 22]. One-hundred and nineteen students (32% of the survey respondents) expressed an interest in participation in a follow-up interview study (open to all) which would explore topics addressed in the survey in more detail. Fifteen interview participants aged 16–22 years (mean age 17.4 years) were recruited to the final study. All, except one were female. Ten (67%) described their ethnicity as white, with the remainder having a mixed ethnic heritage. Survey participants who went on to take part in the interview study (in comparison to those who chose not to) were more impulsive on measures of negative urgency (but no other UPPS-P variable) and had higher emotion dysregulation and anxiety, and intolerance of distress (all p < .05). (See Table 1 for details of participant characteristics and self-harm presentations). Participants were provided with an opportunity to take part in a draw for a £25 voucher as a thank you for participation.

**Table 1:** Participant characteristics and self-harm status as provided in original survey and interview study.

			Survey-based responses												Interview study	
			Impulsivity mean score								Self-harm				self-harm	
Pseudonym	Age	M/F	NUR	LPS	LPM	SS	PUR	DERS	DTOL	HADS Depression	HADS Anxiety	Lifetime presence	Frequency	Recency	Recency	Method
Annie	*17*	F	11	5	7	12	11	51	2	7	10	yes	Rarely	current	current	punching
Bella	*22*	F	16	12	8	12	11	62	1.5	9	12	yes	Often	6 months	current	cutting/biting
Caitlin	*17*	F	7	8	6	12	5	56	3	8	11	no	-	-	-	(outbursts)
Dionne	*18*	F	15	7	12	10	8	70	2.5	13	19	yes	Often	current	6 months	severe scratching, punching walls
Elizabeth	*17*	F	12	9	10	6	11	65	1.5	8	11	yes	Very Often	Current	current	cutting, punching something
Fleur	*16*	F	14	10	12	12	11	66	1.9	7	17	no*	-	-	current	swallowing substances
Grace	*17*	F	4	7	4	6	4	47	3.4	1	5	yes	Very Often	> year	> year	cutting
Helen	*16*	F	9	7	5	8	8	37	3	2	6	yes	Very Often	> year	current	cutting
Ivy	*16*	F	11	6	7	9	6	57	3	3	11	yes	Rarely	> year	current	(Disordered eating)
Jen	*17*	F	13	6	9	13	8	66	2.4	10	15	yes	Sometimes	> year	> year	cutting (binge eating)
Karl	*17*	M	10	6	6	13	9	58	2.6	13	9	yes	Rarely	> year	6 months	cutting
Laura	*19*	F	15	9	10	10	11	78	1.4	11.5	10	yes	Very Often	2 months	6 months	cutting, burning, hitting
Mel	*18*	F	9	8	5	13	14	58	3.5	7	12	yes	Very Often	6 months	> 2 years	cutting
Nicole	*17*	F	9	5	6	9	4	59	3	6	15	no*	-	-	> year	punching walls
Olivia	*17*	F	12	8	9	11	8	63	2.8	7	18	yes	Sometimes	2 months	current	Scratching, punching walls
Mean (SD)	*17*.*4 (1*.*5)*		11† (3.7)	7.5 (1.9)	7.3 (2.5)	10 (2.4)	8.6 (2.9)	59.5† (9.8)	2.5† (0.7)	6.9 (3.5)	12† (4.1)					
Survey mean (SD)			*9*.*4 (3*.*15)*	*7*.*8 (2*.*3)*	*7*.*9 (2*.*2)*	*10*.*6 (3*.*0)*	*8 (5)*	*48*.*1 (13*.*7)*	3.17 (2.56)	*8 (7)*	*5 (5*.*7)*					

Notes:

* indicates that participants reported a qualitative change in behavioural endorsement of self-harm in the interview.

Frequency of self-harm: Rarely (1–2 times); Sometimes (3–5 times) Often (5–10 times); Very Often (>10 times). Recency: Current (past 4 weeks); 2 months (within the past two months); 6 months (within the past 6 months) >year (over a year ago); UPPS-P Impulsivity scale (Whiteside & Lynam, 2001; Cyders & Smith, 2007) NUR (Negative Urgency); LPS (lack of Perseverance); LPM (lack of Premeditation); PUR (Positive Urgency); HADS -Hospital Anxiety and Depression Scale—(Zigmond, 1983); DERS = Difficulties in Emotion Regulation Scale (Gratz & Romer, 2004).

† indicates independent samples t-test revealed a statistical difference in measure scores between the interview sample and scores from those who did not take part in the interview p < .05).

### Measures

Face-to-face sessions were structured around two card-sorting tasks and a semi-structured interview. Participants were invited to talk about psychological processes and self-harm, but if individuals chose not to talk about self-harm, or were unable to, we would seek to understand the interaction between these ideas and other potentially risky or harmful behaviours as identified by young people. Data were generated through open-ended questions, follow-up probing, prompts and clarification-seeking, which enabled a flow of conversation throughout the entire face-to-face session.

#### Card sort Task 1 –“All about me”

**Fig 1:**
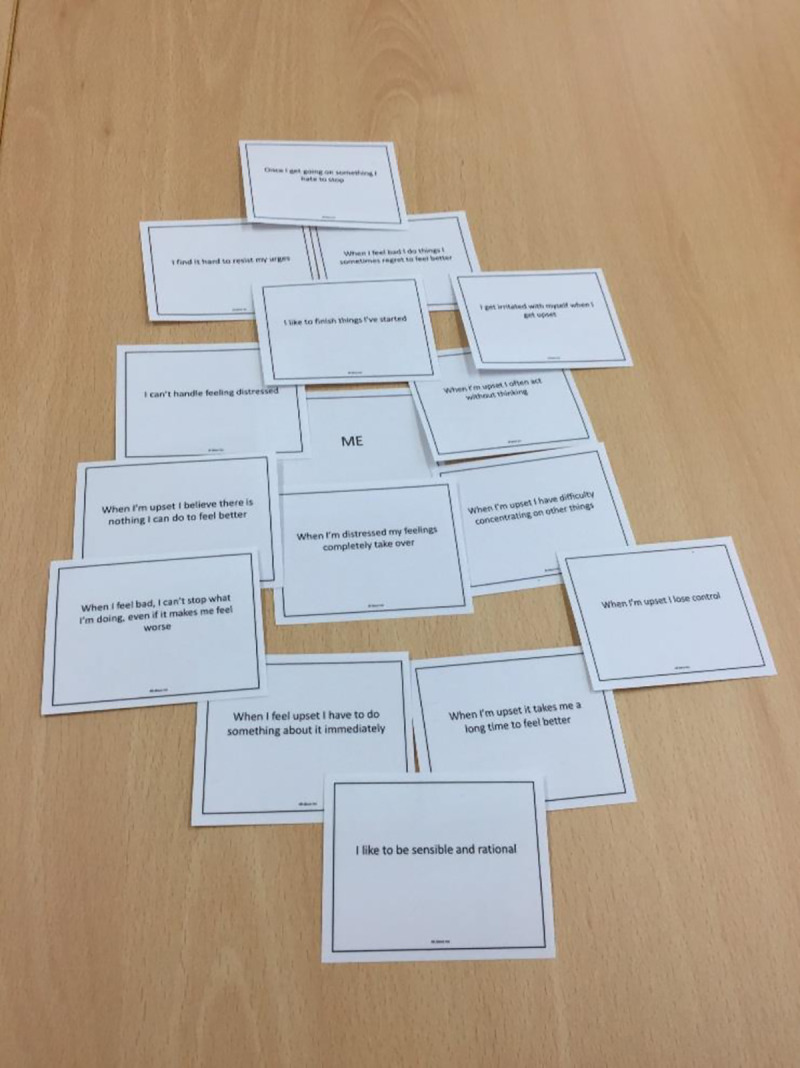
All About Me card selection.

Task 1 facilitated discussion of the ways in which trait-based items relating to impulsivity, emotion and self-regulation were perceived by individuals to be characteristic of their own personality. Twenty-five cards displayed items from self-report measures examined in the original college-based survey. Items with higher mean scores for each measure in survey responses were selected (judged as representing strong endorsement of the criterion of interest). Increased weighting of cards was given to measures representing impulsivity and rash response to emotion given the scope of the present study, and items were chosen across measure sub-scales. Cards comprised: 11 items from UPPS-P [23] which assesses five distinct personality-traits that lead to impulsive behaviour; seven items from the Difficulties in Emotion Regulation Scale [24] which assesses clinically relevant emotion dysregulation; three items from the Distress Tolerance Scale [25] which assesses the extent to which an individual believes that they can accept and withstand distressing emotional states; and four items from the Brief Self-Control Scale [26] which assesses individual differences in the ability to control one’s behaviour. Participants were given the full set of cards, a “Me” card, and additional blank cards for participant-generated items. They were instructed to select and arrange relevant cards around the “Me” card. Participants were informed that this task was a way to explore some of the ways they might think and feel, and that there were no right or wrong answers. Once the selection had been made the researcher facilitated a discussion around the choice and position of cards (See Fig 1).

#### Card sort Task 2 - “My experience”

**Fig 2:**
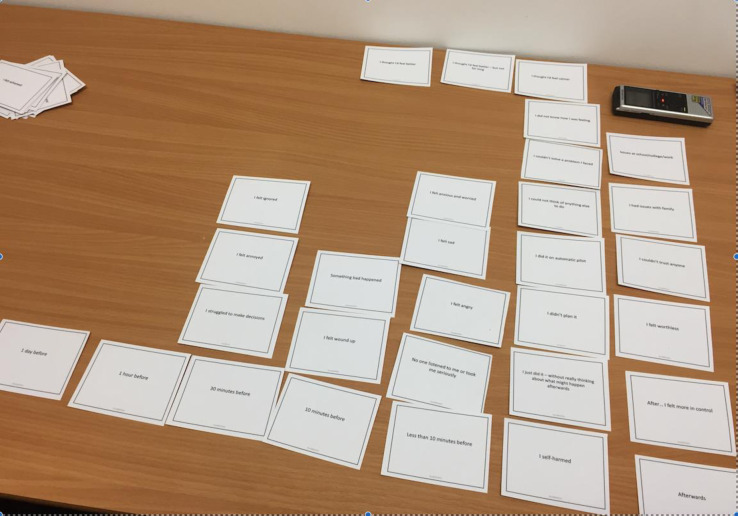
My Experience card selection.

A modified version of the CaTS [18] explored factors identified as relevant by participants in the moments leading up to and following a self-harm episode (or other impulsive action). Participants were provided with a set of cards 43 cards describing thoughts, feelings, events and behaviours. These included: 30 items from the original CaTS, which aligned with the focus of the present research (e.g. *I could not solve a problem I faced*); eight additional items identified in open responses in the survey (e.g. *I felt wound up*); and five items which captured the expectations that might be associated with a self-harm act (e.g. *I thought I’d feel better*). Additional blank cards were provided. In the original study participants arranged cards along a timeline, which ran from 6 months before self-harm to after self-harm had occurred. In the present study we compressed the timeline given the evidence that youth who self-harm often act within 10 minutes of first thinking of self-harm [27]. Thus, the modified timeline ran: One day before, 1 hour before, 30 minutes before, 10 minutes before, less than 10 minutes before, 5 minutes before, At the moment of self-harm (or other behaviour), and Afterwards. Participants were asked to think about a specific time when they had self-harmed (or other problem behaviour) and had good recollection of the experience. They were asked to choose cards most relevant to this experience and place them along the timeline in order of occurrence. The researcher facilitated a discussion around the choice and position of cards, during or following completion of the task as preferred. In some cases, cards were moved into multiple timeline positions (Fig 2).

#### Interview schedule

A semi-structured topic guide included questions concerning individual history with self-harm; questions relating to the card sort tasks; and questions relating more broadly to facets of impulsivity. However, the approach was flexible to allow for wider discussions around emotion, impulsivity and self-regulation where identified by participants. Topics explored were informed by previous research and theoretical understanding about impulsive pathways to problem behaviour, and by open responses obtained from the earlier FE survey. (See S1 File)

#### Procedure and analysis approach

**Fig 3:**
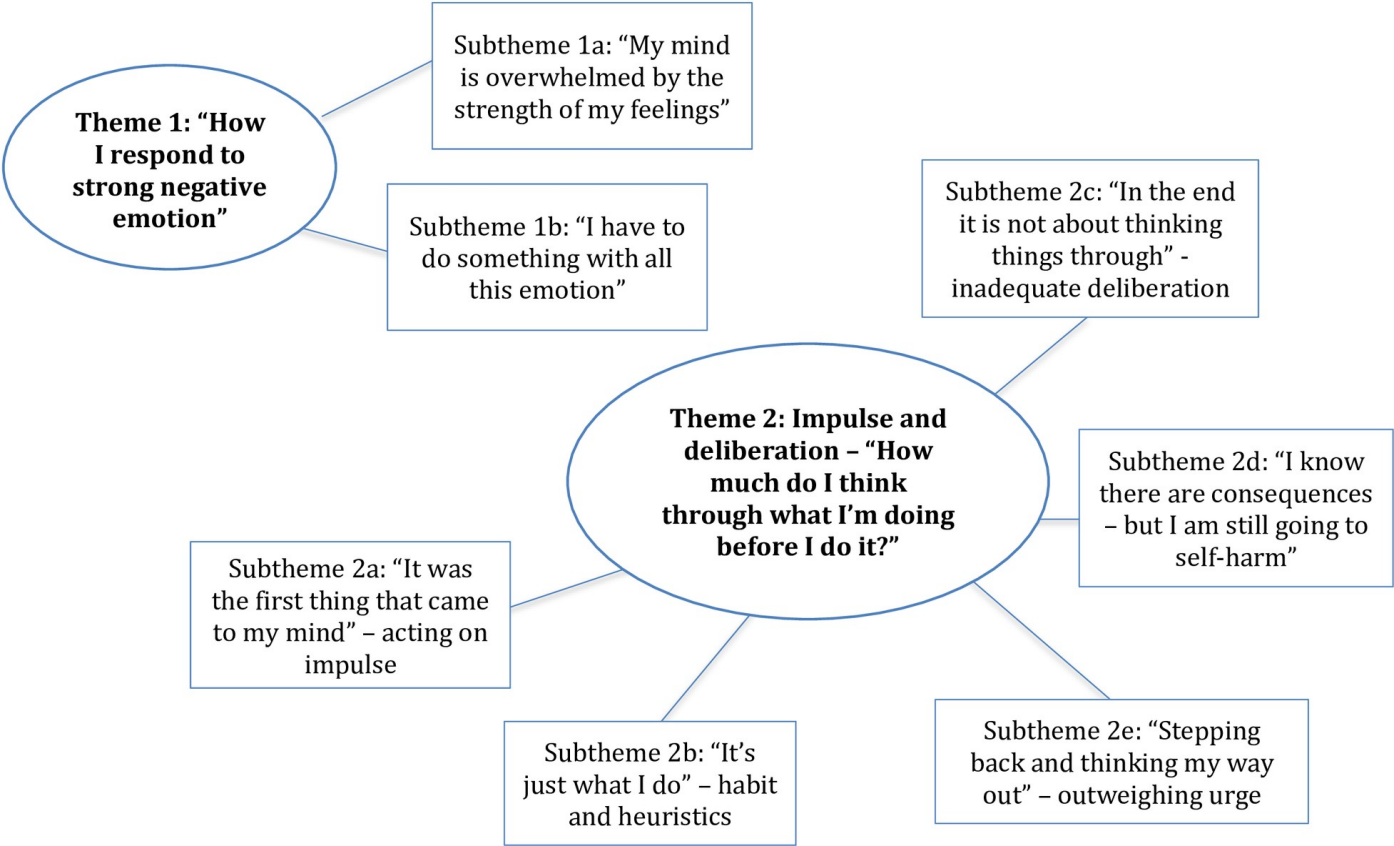
Thematic map showing main themes and subthemes.

Participants over 16 years of age provided informed consent in accordance with Ethical approval obtained from the Division of Psychiatry and Applied Psychology Research Ethics Sub-committee at The University of Nottingham (Ref 243). Sessions were conducted between September and December 2017 at college or University locations according to participant preference. Sessions were audio-recorded, transcribed verbatim and anonymised. Sessions lasted from 45.13 to 56.02 minutes. As part of participant wellbeing checks, a pre-post session emotional rating score using a simple visual analogue scale (VAS) was completed. The researcher engaged participants in conversation about changes to mood status over the course of the interview and provided signposting support. A final mood elevation exercise was also included. Data were analysed by the first author according to Thematic Analysis guidelines [28]. Themes were extracted inductively (as derived from the data) and deductively (on the basis of past evidence and theory). A coding framework identified conceptual ideas based on self-harm, emotion and impulsivity, other internal processes of self-management, and developmental processes. Transcripts were read in turn to note interesting features and develop initial codes according to the framework. Additional codes were included when novel, compelling areas of interest were identified. Transcripts were read multiple times to ensure all relevant patterns and meanings in the data had been considered, codes were then organised into potential themes. A process of review and refinement took place with themes judged according to individual coherency and representativeness of the dataset. Following revisions and refinements, two overarching themes and additional subthemes (Fig 3) were derived. A summary codebook was created which included theme descriptions and exemplars. Inter-coder reliability between an independent researcher and lead author based on data extracts using the codebook were assessed using Cohen’s kappa and demonstrated substantial agreement *(k =* .*74 p <* .*001)*. The first author is a mature, female qualitative researcher and youth counsellor who recognises that her position may have shaped the power dynamics in the research relationship and the subsequent interpretation of data. A reflective diary facilitated an active and continual reflexive engagement throughout the research process and sought to counter subjective bias. In addition, the card-sort methodology attempted to bridge the power differential by providing tasks in which young people held agency and greater control over the direction of conversation.

## Results

Participant characteristics are presented in Table 1. During the interview, two participants disclosed self-harm behaviour which had not been indicated in the original survey. In each case, participants suggested they were more comfortable discussing self-harm in person. All participants described having had thoughts about self-harm. Fourteen participants endorsed self-harm and thirteen reflected on an episode of self-harm during the card-sort tasks. One participant (Ivy) endorsed self-harm but chose to reflect on the build-up to a recent episode of binge eating. One remaining participant (Caitlin) did not endorse lifetime self-harm. She reflected on harmful impulsive and anger driven outbursts during the card sort task and discussed combatting urges. The insights of these participants during the two card tasks and wider interviews were considered informative about the emotional and regulatory processes underpinning rash behaviour and the role of impulsivity in the transition from thought to act and hence of empirical value to wider discussion around the emotional, developmental and cognitive context of problematic behaviour in youth. However, these accounts did not contribute to focused analysis of the build-up to self-harm which is based on thirteen narratives.

Card tasks were primarily used to facilitate discussion and scaffold understanding, nonetheless, the frequency of card endorsement revealed a pattern of thinking and acting which categorises a group of young people who describe risk-taking behaviours and endorsement of self-harm. In Task 1, high frequency items reflected both dispositional difficulties in managing and tolerating negative emotion (*I get irritated with myself when I get upset*), but also high Perseverance and Premeditation i.e. low impulsive, controlled and rational behaviour, (*I like to finish things I’ve started*; *I like to be sensible and rational*). Some participants commented that their choice of card might reflect who they wanted to be or how others perceived them. Additionally, young people suggested that they recognised themselves as having multiple, and changing characteristics determined by situation and peer influence. In Task 2, items relating to anger and annoyance were strongly endorsed across multiple time points. Items relating to emotion driven response and dysregulation and deficits in cognitive and problem solving were important precursors in the build-up to behaviour, but deficits in conscientiousness were consistently identified as most proximal (See Table 2). In Task 2 five young people chose the card ‘I wanted to die’ in relation to the episode recounted. In three cases, young people clarified that this was not about suicidal intention, but reflected a desire to achieve a state of ‘not being present’ in the pain of the moment (Elizabeth); a recognition of ‘hopelessness’ (Fleur); or reflected a ‘dormant’ feeling that suicide would always be an available option (Laura). One participant chose this card as being relevant in the moment of self-harm (Mel) but described this as a way of ‘getting rid of upset’ not a planned intention to die. When probed, Dionne, who also selected this card, described being confused about her motivation. One participant (Elizabeth) selected the card ‘It stopped me from killing myself’ after self-harm and suggested that this was about behaviour providing a means of release from the intensity of emotion that might escalate if left unchecked, but not about risk in that moment. Participants who endorsed cards relating to wanting to die varied in their presentation of self-harm amongst each other, and within the sample did not appear to have a distinct profile.

**Table 2:** Card-Sort Task 2 (My Experience) showing frequency and item selection according to time stamp for those endorsing self-harm.

Frequency	Items relating to	One day	1 hour	30 mins	10 mins	<10 mins	5 mins	I self-harmed	Afterwards
33	Anger/annoyance	5 (6)	6	6	4	5	3	2	2
31	Feeling worthless/burden/hating self	2	8	4(6)	4(5)	3	4	1	5
29	Poor problem solving/ executive functioning deficits	4	4(5)	5(6)	5(8)	5(6)	3	3	
27	Life events (family/friends/boyfriend/girlfriend issues)	16	7(9)	3					1
25	Feeling ignored / rejected /not listened to	5(6)	1(4)	6(7)	2	4	1	3	3
25	Acting without planning or thinking through consequences			1	4(6)	1	2	15	2
20	Feeing sad / hopeless	2(3)	4	6	3	1		3	1
17	Expectations that would feel better/calmer/more in control	1	2		2	2	1	5(6)	4
13	Thinking about something bad happening	3(4)	4	2	4				
10	Feeling anxious / worried	1(2)	4		3	1			1
10	Acting rashly in response to emotion	2			1	2		5	
11	Feeling better/calmer/more in control after self-harm	1							10
8	Feeling numb		(1)	1	1		1	3	2
7	Feeling worse after self-harm								6(7)
5	Feeling ashamed	1			1				3(4)
5	Wanting to die		2	1	1			1	
4	Not being able to trust anyone	1	1(2)						2
5	Having access to the means to hurt myself	2	1			1	1		
4	Not expecting to feel any different			1		2			1
4	Not feeling different after self-harm								4
1	Self-harm stopped me from killing myself							1	

Notes: Table presents card selection of 13 narratives which specifically detailed the build-up to self-harm in Card-Sort Task 2. Item frequency is shown in brackets when all 15 participant item selections are included. Life events were highly endorsed distal (one day) precursors to behaviour. Action without planning or deliberation were highly endorsed proximal factors at the moment of behaviour. Items relating to positive change in feeling/control were most consistently endorsed following self-harm.

The thematic analysis draws on the narratives of participants captured across the whole face-to-face session (card-tasks + interview) and draws out themes relating to the salience of impulsivity and the wider emotional/cognitive context as a distal/proximal influence in relation to self-harm thoughts and the build-up to behaviour.

### Overarching theme 1: How I respond to strong negative emotion

How young people recognised, experienced and responded to strong emotion was a key component of interview narratives. This overarching theme encompassed the idea of an emotional build-up that is poorly tolerated and precipitates cognitive and behavioural response. We distinguish ‘emotion’ (as a specific, action-oriented response which may be brief in duration) from ‘affect’ which captures a range of feelings encompassing specific emotions and a less-defined mood [29].

#### Subtheme 1a: “My mind is overwhelmed by the strength of my feelings”

Participants described themselves as being emotional in general, often experiencing feelings of anger and annoyance, but also sadness and anxiety. This negative emotional context was complex, characterised by multiple, simultaneous and pervasive feelings. For many, these emotions “escalated” or “intensified” until reaching a pressure point. Young people conveyed the difficulties that they had in tolerating this emotional presence. This was not about problems in emotional awareness or identification, but about reaching a threshold of emotional intensity beyond which they felt overwhelmed. Young people described cognitive responses to this emotional overload. Some described a narrowed fixation on their emotionality,

*“I have difficulty concentrating on things other than the fact that I’m really*, *really*, *upset or angry”* (Helen). “*It’s hard to focus on anything else when I have those feelings*” (Elizabeth).

For others, the perceived inability to deal with and contain negative emotion was the root cause of further emotional response. Being emotional was a failure in self-control and thinking about that failure provoked an escalation in negative emotions, generally anger and frustration. “*It irritates me that I can’t control getting over-emotional*” (Grace). During Task 1 all participants chose the card *I get irritated with myself when I get upset*, overwhelmingly identifying this as characteristic of their typical thinking. For Dionne, this self-directed annoyance conveyed an anxiety about being unable to prevent the return to previous over-emotionality. Hence, emotionality was tied up with cognitions of how she used to be,

“*Whenever something does upset me it reminds me of when I used to get really upset and I’m like ‘no*, *no*, *no*!*’ so I kind of almost start to panic*. *So whenever I feel anything that’s not ‘oh*, *I’m alright today’*, *or ‘I’m happy today’*, *I’m a bit like ‘Oh God*, *oh God*, *oh God…’*”

Hence, emotional response is amplified by cognitive processing. Consistently, young people not only experienced heightened emotion but also perceived themselves as having a reduced capacity to “handle” those emotions. All participants except one (Nicole) selected the *I can’t handle feeling distressed* card in Task 1 and identified with a notion of poor distress tolerance. Mel described past risky behaviour in which she felt so unable to deal with negative emotions that she would choose to relinquish responsibility for her emotions to others. She described crossing roads with her eyes closed to “*let someone else decide*”. (Notably, Nicole suggested that while she could handle her response to distress and sadness, she did not know how to respond to anger.)

#### Subtheme 1b: “I have to do something with all this emotion”

Participants described how a “build-up”, “escalation” or “intensifying” of emotion often reached a pressure point that precipitated a behavioural response, usually self-harm. Anger was frequently the catalyst for action, with participants describing a desire to take their anger out on something, or someone–typically themselves. Anger, for many was considered likely to result in an immediate behavioural response. Items relating to feeling “angry” or “wound up” were selected at every time point before self-harm by one or more participants (Table 2) and this emotional response was perceived as an important proximal trigger precipitating a self-harm episode. Two participants suggested that an anger-related ‘explosive’ self-harm which drives an immediate reaction is a qualitatively distinct variant of self-harm:

“*the explosive angry rage one*” (Laura); “*the explosive kind…the hair pulling*, *biting*, *here*, *now*, *do something*, *kind”* (Bella).

Research has indicated that the transition from thought to act may be linked to the elevated arousal associated with an anger state [30]. Associations between anger/aggression, impulsivity and self-harm are documented in clinical and research samples and it has been argued that an additive relationship between anger and impulsivity may serve to intensify the likelihood of quick behavioural response [31]. Collectively, these accounts underline the importance of clarifying the specific nature of emotion and its likely behavioural response in relation to self-harm. Participants described the benefits of a quick removal of emotion with self-harm. For Nicole this was about regaining cognitive control, *“I need to change it [the emotional build-up] quickly*, *because then I can focus on other things…”* (Nicole). Jen also recognised the need to do something with an emotional build-up immediately, otherwise “…*it will just drag on and it will make me feel worse*”. At ten minutes before self-harm she articulates the anxiety she felt being in the midst of a spiral of emotion and cognition, aware she would need to ‘do something’ with the emotional load,

*“I was like*, *what’s the next step*? *What am I going to do with all these feelings*? *Am I going to let it go*, *keep it to myself*? *…what will I*, *like*, *what would I do to myself*?*”* (Jen)

At this point, she suggests this could be self-harm or something else, but as her rumination increased (not about the original trigger but about how to manage “all these feelings”) she describes just needing to act, *“I’d just go do it…because if I don’t do it*, *I will do something worse”*. (Of note, a similar presentation of a response to emotional pressure was discussed by Ivy in the context of the lead up to an episode of disordered eating where a build-up of pressure and rumination led to an act.) The narratives underscore that emotion and cognition work in concert to determine behaviour response but recognise that the rapid response to emotional pressure with a ‘known behaviour’ that allows a quick and predictable outcome, was seen as beneficial and not rash.

### Theme 2: Impulse and deliberation—“How much do I think through what I’m doing before I do it?”

Different and often competing systems of behavioural determination were present in young people’s accounts of the processes leading to self-harm. These systems related to impulse-driven or automatic behaviours, or to processes that were more deliberative, and mindful of potential consequences. Arbitration between these modes of behaviour was evident.

#### Subtheme 2a: “It was the first thing that came to my mind”—acting on impulse

A mode of response characterised by quick action without much, if any, additional thought, was identified at some stage of their personal stories by almost all participants and was typically identified within ten minutes of self-harm:

“I would just go to the toilet and punch a wall until my hand was bleeding. I didn’t think about it, I just walked out of class and went.” (Annie). “I kind of didn’t think about it, but just wanted to let my anger out and it was the first thing that came to my mind, so I just went for it.” (Nicole)

Items relating to action without planning or deliberation were strongly endorsed cards in the moment of self-harm (Table 2) and correspond to the idea of an irresistible-impulse model of self-harm [4]. Some young people suggested that the impulse to self-harm appeared to take them by surprise, or just happen “out of the blue”. Fleur, who described drinking bleach during one episode of self-harm, explained,

“A lot of things I went through on impulse… even with the bleach, that was completely–I wasn’t even thinking about it two minutes before that. I was washing up.”

This resonates with escape models of suicide and self-harm [32] which describe impulsive acts occurring in a moment of cognitive deconstruction where self-awareness is removed, temporarily overridden by the drive for short-term gratification. This mode of response is typical of impulsive pathological behaviour (see [33]). Annie’s recollection of punching a mirror at college until her hand was bleeding and the mirror broken, only surfaced when a staff member questioned the tutor group days later. Others similarly described being “confused” to find that they had self-harmed, or unable to remember any details about the act. Arguably, attempts to circumvent an urge to self-harm may then involve overriding this impact on executive resources. The challenge of restraining a behaviour that is being performed outside of conscious awareness is succinctly identified by Fleur,

“Whenever [the impulse to self-harm] happens to people, they don’t know what they’re doing… maybe we need to see the warning signs first. Because once it gets to the urge, maybe it’s too late.”

By contrast, three participants described the ability to delay the expression of an urge to self-harm until a convenient occasion arose to gratify it. Elizabeth described not necessarily having the necessary equipment when the urge to self-harm occurred and so “keeping those feelings”; Bella described simply putting the impulse off if she needed to. Grace described delaying the impulse until a more appropriate time and suggested that passage of time or change in environment did not dampen her desire to self-harm, rather, containment of the urge–in the knowledge that it would be addressed at a later stage–enabled it to be retrieved at its original state of urgency. Theorists have discounted that acts of suicide can be ‘impulsive’ on the basis that they are rarely undertaken without planning, and that consideration of an impulsive act of suicide may have occurred in the hours, days, or weeks before an eventual act [16]. The present accounts point to a temporal disconnect between having an urge to self-harm and acting on that thought, such that thought is subsequently unnecessary before an act. Hence, while these participants suggested that their self-harm occurred on the spur of the moment, prior consideration may suggest that these are not impulsive acts. Notably, all three endorsed more established frequency of self-harm. The behavioural profiles of individuals able to delay an impulse to self-harm could potentially result more from a problematic level of over-controlled behaviour, which suggests a different risk profile to someone acting purely in terms of an irresistible-impulse.

#### Subtheme 2b: “It’s just what I do”—habit and heuristics

The notion that self-harm was a habitual response was present in several accounts. A number of participants described an antecedent-consequent logic,

“It’s sort of like an inbuilt thing now. It’s like…I’m feeling like that, so then, I’ll do this [self-harm]” (Elizabeth); “It’s like Maths–you add ‘this’ and then ‘that’ and it’s equal to self-harm” (Jen).

Narratives gave the sense of self-harm as a heuristic–a learnt association, strengthened over time and experience, and easy-to-access as a go-to option in the moment of distress,

“So, I think it’s very much what will help me immediately–oh, I’ve self-harmed before, I’ll do that again.” (Olivia).

A reliance on affect-based heuristics—decision rules based on affect—is a typical adolescent response, which has been shown to increase risk of maladaptive behaviours [34]. The adoption of a simple decision rule–this made me feel better when I felt like this last time, therefore I’ll do that if I feel like that again–does not require careful consideration. As such, this mode of processing information is not compromised by potentially immature cognitive control systems characteristic of adolescence. It is suggested that impulsive behavioural systems may operate in a low-cognitive mode in which automatic responses built via associations learnt and stored in long-term memory, can be reactivated quickly [35]. As such, self-harm behaviour may offer young people a quick and known response option without the cognitive costs of thinking through an alternative course of behaviour or using cognitive resources to try to inhibit or override a pre-potent response. In two cases, where young people described frequent and current self-harm (Mel and Laura), they described habitual behaviour as something ingrained, as, “Just something that you feel like you have to do.” (Laura)

#### Subtheme 2c: “In the end, it is not about thinking things through”- inadequate deliberation

Participants directly explored the notion of self-harm occurring–or not–following careful thinking and reflection of potential consequences. For some the decision to self-harm was not planned but proceeded in the absence of any deliberation,

“I would have done it [self-harm] as soon as I thought of it…I wouldn’t have considered any consequences. I would just have done it.” (Olivia)

However, others indicated that they did engage in active deliberation or recognised the consequences of behaviour but described that deliberation as unsuccessful,

“I try and really think about things before I do them. But then it’s like–I find it really hard to resist urges” (Dionne); “It’s usually when it comes to the point like I can’t find any reasonable solution… then I just can’t stop myself from getting to the point of self-harm.” (Helen)

Potential difficulties with executive functioning during self-harm episodes, are reflected in the items selected in Task 2. The card “I could not think of anything else to do” was the most frequently endorsed card in the 10 minutes before self-harm. Other items relating to cognitive components in the moments leading up to and at the point of self-harm (“I couldn’t solve a problem I faced”; “I struggled to make decisions’) were also recognised as proximal risk factors. As such, narratives indicate this may be less about a failure to delay action in favour of careful thinking, but about the inadequacy of that deliberation. By the moment of self-harm, the urge had become too strong to be outweighed by deliberation. For Laura, the effort of resisting the urge to self-harm over a long period of time meant that at this point she “didn’t care” about the consequences anymore and would “just do it”. This resonates with the strength model of self-control where impulsive behaviour may result from a depletion in cognitive capacity [see 36] and a wearing down of cognitive reserves through continued attempts at resistance.

Narratives suggest there may also be an interplay between forward reflection and perseverance i.e. an inability to pursue or stick with the identified alternative course of action. Given that the (lack of) Perseverance item ‘when I get going on something I hate to stop’ was the most endorsed card in Task 1, selected by all participants, it is possible that high persistence (e.g. self-discipline and will-power) could play a role in maintaining continuation with self-harm as the chosen course of action. Consistent with this position, two participants saw cognitive difficulties with distractibility and indecision (low persistence) as protecting them from acting on their impulses to self-harm.

#### Subtheme 2d: “I know there are consequences–but I am still going to self-harm”

Not all accounts fitted into a pattern of behavioural determination where self-harm resulted from inadequate reflection or a failure to carefully consider outcomes and consequences. In some narratives, young people suggested that they fully recognised and acknowledged the consequences of action, but pragmatically dismissed them,

“I know about the consequences and stuff, but I just do it anyway–the consequences are very much there” (Helen); “It’s weird because it’s like, you understand the consequences and you know that it’s not right, but you do it anyway” (Laura). “I could always see the consequences of what was going to happen, which ultimately was I'd patch myself up, I’d get on with my day, and eventually it would heal. Umm, so there weren't any consequences that I couldn't handle” (Grace).

Notably, high tolerance for negative consequences of behaviour is consistent with trait (lack of) Premeditation [37] where perceived negative consequences may be insufficient to deter behaviour. Nonetheless, these accounts highlight the granularity necessary in understanding how behaviour relates to deliberation. Importantly, some participants described positive outcomes of self-harm. For these young people, self-harm was logically motivated and they welcomed the consequences e.g. as a way of gaining positive attention or support, feeling more in control, or just feeling something. These narratives reinforce that in the absence of self-harm an alternative means of achieving this function would be necessary.

#### Subtheme 2e: “Stepping back and thinking my way out”—outweighing urge

In many instances, where young people were able to avert a self-harm episode or another rash behaviour, they described employing a range of higher order mental operations that allowed them to stop and question and ultimately outweigh urge. These included: being able in the moment to focus on the “bigger picture”; to think about how the decision to self-harm would impact on another; to plan an alternative course of action; to wait for urge and emotion to naturally “subside”; to reflect on the short-term pain and inconvenience of caring for wounds, or the long-term impact of scars; and to focus on workable targets or long-term goals. Endorsement of protective, reflective processes over emotion-driven impulses were particularly evident in the histories of young people who reported no longer self-harming. Some described a changed emphasis in conscious and deliberative effort over time suggesting that they were now better able to take a step back from the emotional reactivity and recruit effective techniques of internal management. For some this change was a result of maturity and additional responsibility that comes with age,

“I think as I’m getting older, I’m trying to be rational about things, and trying really hard to be sensible, like you shouldn’t just work on impulse” (Dionne); I’m trying to be like, be an adult, and I'm like, I feel like, I have little tolerance when I'm acting out because I’m like–‘You're not 16 anymore, like stop!’” (Laura).

The process of being rational and deliberative remains an effort, but the motivation to control impulses has changed. While most self-harm behaviours in adolescence resolve spontaneously over time [38], these accounts outline the involvement of personal determination in this process. Moreover, they suggest that where young people describe impulsive pathways to behaviour and acting without premeditation, these are not perceived as immutable traits. Other participants suggested the change in behaviour from someone reliant on self-harm to someone who is not, was about learnt experience,

“I came to recognise that there's always a pattern with it—it's just you get over-excited or just really upset and then you just, you know, act irrationally. So now I can kind of stop myself and ask, ‘ok, why do you feel like that?’ and then think about it, rather than just go crazy” (Mel).

This reflective, adaptive capacity was described by those who thought about self-harm but had not acted on the urge. Caitlin, who described herself as impulsive, articulated how she keeps impulses under control,

“So I think, ok, I’m in this mood, it’s late, my parents aren’t at home, and I’m quite emotional, and I know that if my emotions sort of dominate the other sort of rational side of me, then I know that’s when you’ve probably got a bit of an issue…so I make myself go outside, or make myself do something”.

## Discussion

This study sought to gain an understanding of the experience of self-harm for young people in relation to facets of impulsivity and broader processes relating to emotion and self-regulation, and specifically to consider the salience and interplay of these factors in the hours and minutes before an individual episode of self-harm. The novel card-sort methodology sought to facilitate understanding and nuanced discussion.

Overall, participants reported that being impulsive was a common characteristic of their personalities and often a strong behavioural feature in the short-term build up to a self-harm act. Rash response to heightened arousal, often enmeshed with poor cognitive management and difficulties with behavioural control, created a context for self-harm that resonated with many, with anger a typical accelerant of an emotional-cognitive spiral towards behaviour. For some, the tendency to act rashly without due regard for consequences intensified risk. Others reflected on consequences, but deliberation was insufficient to outweigh urge; or described acting on the basis of quick go-to responses that did not require deliberation. However, some described behaviour that could be controlled, deliberative or perceived as rational and not rash. Overall, participants described a tension between impulse and control, the equilibrium of which was dynamic and appeared to change as a function of age and maturity. As such, modes of response were judged by young people to be unfixed and targetable.

### Strengths and limitations

The study provides novel qualitative insight into the links perceived by adolescents between their own behaviour and psychological processes that focus on impulsivity, emotional response and self-regulation. Evidence has shown that many young people act within 10 minutes of first thinking of self-harm [27] including 40% of survey respondents who said they acted within 10 minutes of first urge. In addition, research suggests that acting on impulse is an immediate pre-cursor to self-harm [18]. As such rich information may be gained by examining the interplay between psychological factors in the moments prior to engagement in self-harm with young people. Participants identified with discrete facets of impulsivity (as delineated by the UPPS-P model) as meaningful characteristic traits relating to self-harm, or other risk-taking. The work thus extends the discriminative utility of the UPPS-P to a qualitative context. However, findings also provide insight not only into the endorsement of impulsive facets, but into how these pathways to behaviour were interpreted and loaded. For example, as a means of stemming emotional escalation the ability to act quickly without careful planning and forethought was recognised as a beneficial protective factor; and easily accessible response heuristics which do not require careful consideration in the moment and are not therefore compromised at times of stress, are seen as rational and logical. The tendency to experience strong impulses in the context of heightened emotion (consistent with Negative Urgency) or to act with low conscientiousness (consistent with lack of Premeditation or Perseverance) were endorsed, but not necessarily interpreted as rash or problematic. It is important to recognise this nuance in discussions with young people in which impulsivity might be framed as a problem behaviour.

The novel card-sort task framework was successful in facilitating discussion and personal awareness and supporting young people to articulate complex patterns of thoughts and behaviours. This is significant given concerns that youth may struggle to recognise or identify the cognitive processes that underlie actions [19]. The ability to visually and physically review and manoeuvre cards, creating patterns between items, appeared to help young people pinpoint and describe salient factors while clarifying personal understanding, and was an important boon to exploring complex phenomena with young people. One participant’s appraisal of the process was that it, “just kind of makes me realise how messy it all is, like, just feeling so much at once”. She found it helpful in normalising and thinking through that complexity. Our, findings therefore support the research utility of card-sort methods for self-harm in youth within qualitative work [18]. Using this approach in clinical settings with individual clients to explore the cognitions driving self-harm would be a useful extension. Importantly, the study design helped to reduce the power differential between participant and researcher by providing tasks that established rapport and grounded the participant in tasks in which they were the expert and controlled the direction of discussion. This power redress is particularly pertinent for research in education-based settings that flout the nature of power symmetry [39]. Moreover, it provided a research task which young people reported enjoying and valuing.

These strengths should be considered in the light of some limitations. Firstly, the sample represented a small and largely homogenous group i.e. predominantly female, FE college-based students with a history of self-harm. This was a convenience sample derived from a larger study with increased gender diversity and experience, and despite wide initial interest, challenges in recruitment limited the diversity of the final sample. While the female predominance limits the generalisability of the findings, it is recognised that a substantial and increasing portion of those reporting experience with self-harm thoughts and acts are young females [1, 40]. Nonetheless, care should be taken to explore the endorsement of risk factors identified in the present study with young males. It is also noted that the interview sample reported increased scores against certain psychological variables in the original survey (Negative Urgency, Anxiety and Emotion Dysregulation and intolerance of distress) in comparison to the survey sample overall, and therefore represents a sample biased towards heightened difficulties in responding to emotion. It is important to recognise that in two cases participants chose to discuss different experiences to self-harm when describing the short-term build-up to behaviour. These accounts are informative in the wider context of how psychological factors such as emotion-based impulsivity and emotion dysregulation contribute risk for adolescent rash behaviour, and inform the progression from thought to act, but these accounts do not contribute to a focused examination of the short-term build-up to a self-harm episode per se, and their reflections may qualitatively differ. Work has established the transdiagnostic relevance of constructs such as Negative Urgency across problem behaviours [8, 41]. Comprehensive transactional risk models have additionally sought to explain how risk and maintenance factors at a distal and proximal level interact with broader cognitive processes as precursors to different maladaptive behaviours [42, 43]. Further work to explore shared risk pathways in the aetiology of different problem behaviours is warranted.

The study design, which built on an earlier survey, was informed by pre-identified topic areas, which may have narrowed the scope of the research. The interview schedule included questions that outlined facets of impulsivity (Negative Urgency and low premeditation in particular), which may have influenced participant endorsement of these pathways to behaviour. Previous work has discussed the potential for self-validation of a problem behaviour as ‘impulsive’ by the inclusion of impulsivity items in a survey (or here a line of questioning) [19] i.e. ‘perhaps I am impulsive if the suggestion is that self-harm is an impulsive behaviour’. Consistently, empirical studies have pointed to the importance of Negative Urgency and low deliberation/planning in understanding self-harm in terms of distal and proximal influences [16, 18, 44, 45] and a strength of the study is in providing a qualitative clarification of the relevance of these facets in discussion with young people. Nonetheless this potentially led to a line of questioning weighted towards corroborating a-priori assumptions. A temporal disconnect also existed for participants asked to describe past behaviours (in some cases dating from over a year prior) in moment-by-moment detail. The saliency of a self-harm act may have aided recall and consistent with other studies [3] no participants found recollection of self-harm or other harmful behaviour to be difficult. The methodology appeared to facilitate this process, but nonetheless the accuracy of responses may have been compromised. It is noted that there were discrepancies in reporting between survey and interview. In part, discrepancies appeared to relate to reduced reluctance to report behaviour in person. This finding may illustrate that a methodology which relies upon a researcher-participant rapport and seeks to ground discussion in tasks which young people themselves could control and take ownership, may be a more suitable method of eliciting disclosure and frank discussion for young people than an online survey. Importantly, it also signals that self-report methods in self-harm studies are prone to inaccuracies, which may stem from multiple motivations.

In our sample participant histories differed in terms of self-harm presentation and across psychological variables (Table 1), with the highest proportion endorsing past month and highest frequency of self-harm. Sub-group analysis work in community-based samples has shown that groups distinguished in terms of frequency, recency, method and function of self-injury differ in psychological profile [46]. Future work exploring short-term build up to self-harm using card-tasks within groups with greater homogeneity of presentation (potentially clinical samples in higher risk categories) is recommended.

### Theoretical and clinical implications

The present findings align with transactional and integrative risk models of impulsivity that combine emotional and cognitive (as well as motivational and behavioural) domains in predicting psychopathology [43, 47] here attempting to examine their meaning for young people in the build-up to behaviour. Integrative models allow for an interaction between impulsivity and broader cognitive processes and cognitions that moderate its expression. Evidence presented here that these processes are not fixed but interact dynamically, underscore that understanding of the role of impulsivity in self-harm should not be divorced from the wider cognitive-emotional context. Furthermore, it is recognised, that the present zoomed in focus on key psychological variables does not reflect the wider, multifactorial complexity of self-harm which results from a fluid interplay of environmental, biological factors and other predispositional vulnerabilities. Card selections pointed to the perceived importance of situational factors (e.g. life events) particularly as a distal (one day) precursor which set a response in motion. Future work could look to examine this wider focus in more detail.

Findings contribute to existing theoretical debates regarding the distal versus proximal relevance of impulsivity to self-harm [15–17] and respond to calls for clearer delineation of trait and state definitions of impulsivity in self-harm research [14]. Facilitated by the card sorting tasks, young people indicated that while they might identify with having an impulsive personality (a distal influence) they also conceptualised their behaviour in the immediate moments before self-harm as action on impulse, divorced of emotion and thinking, a notion close to the idea of an ‘irresistible impulse’ [4] and related to behavioural inhibition as an immediate (proximal) precursor to behaviour. Integrative risk-models of impulsivity have accounted for dual state and trait pathways of influence for impulsivity in explaining maladaptive behaviours such as, for example, eating disorders [42], and may offer trans diagnostic clarification of the connection between these pathways relevant to self-harm. The first pathway relates to momentary impulse, loss of control and a drive to act in pursuit of reward (e.g. arguably, self-harm as a method of affect-regulation). The second pathway reflects trait-based factors, which suggest that stable deficits (such as Negative Urgency) provide a dispositional context for behaviour. Extending such models to self-harm research is a logical step.

Importantly, present evidence points to a period of heightened risk immediately preceding a self-harm episode associated with quick action in response to an urge without much, if any additional thought, and narrowed attentional processing onto self-harm. This proximal risk period represents a critical, if narrow, intervention opportunity (e.g. “Once it gets to the urge maybe it’s too late”) and as such resonates with theorists who suggest that distal, indirect models of the influence of impulsivity on suicidal behaviour offer greater opportunities for support than proximal models [16] given a broader window of opportunity for intervention. However, proximal markers of risk, while narrow, offer more clinically meaningful targets by providing temporal clarity [14]. While there is inconsistent and weak evidence to support the relationship between state-based behavioural markers of impulsivity and self-harm in young people [10] recent work to reconcile the discrepancy between elevated negative urgency (itself a strong indicator of trait based risk) and impulsive action (as a marker of inhibitory control) has indicated that impulsive behaviour in NSSI may involve impaired inhibitory control over initiated negative emotional impulses with behavioural enaction occurring only once urges have surpassed a temporal or intensity threshold [12]. The present findings lend narrative support to this conceptual model. The findings support existing treatment approaches which explore emotional management and deliberative and goal-directed thinking, tackle superficial heuristic processing, and promote strategies to better manage and tolerate emotion(e.g. DBT-A [48]). Present evidence, albeit in a small sample, suggests that helping young people recognise and adopt strategies to deal with arousal at an early stage before reaching their intensity threshold (at which point they may be likely to fall back on response heuristics), may hold value. Card-sort tasks may support the identification of individual ‘warning signs’ and treatment targets.

The Negative Urgency item, ‘*When I’m upset I often act without thinking*’ was identified as a characteristic response tendency by all except two participants in Task 1 and was identified as meaningful in narratives examining self-harm as well as disordered eating, outbursts online, and extreme risk-taking in general, supporting the trans-diagnostic relevance of this trait across psychopathology/problem behaviour [49]. Findings provide contextual support for Urgency Theory [8], which posits that rash action in response to strong arousal is functionally underpinned by brain systems that are biased towards emotion-based stimuli but may lack affective connections to the consequences of actions and long-term implications. Links between adolescence and Urgency Theory are plausible, given maturational differences between established socio-emotional networks and immature cognitive-reflective processes in adolescence [50] and the proposed primacy of the socio-emotional network under conditions of heightened arousal [51]. Evidence presented of inadequate deliberation and a reliance on affect-based heuristics as quick go-to response options operating in a low cognition mode support this developmental framing of risk. Here, late adolescence, by dint of increased maturity, responsibility or learning was associated with greater perceived control over emotional and cognitive processes and the greater likelihood that emotion-driven impulses, while not necessarily abating, could nonetheless be kept in check. Findings underscore that a developmental approach is key to understanding the nature of risk from impulsivity or broader emotional and regulatory processes in self-harm. The development of identity and notions of autonomy is likely to be multifaceted, fluid and changing in adolescence. Identity formation is an important feature of adolescent development and, as articulated by some young people, the selection of cards may have facilitated an alignment with a particular identity. Work to support young people in reformulating negative self-imposed labels (“I am impulsive/indecisive/over-emotional/poor at making decisions”) instead recognising adaptive capability and the potential for self-efficacy in making healthy decisions, or to challenge deficit accounts which reduce impulsivity to a personal failing rather than an adaptive strategy are important implications from this work.

## Conclusions

Qualitative examinations constitute an important addition to understanding of how impulsivity, cognition and emotion might interact in young people to confer proximal risk of behaviour. Adolescents at mid-to-late stages of development with a history of self-harm reported experiencing heightened emotionality and expressed difficulties in tolerating and managing this emotion, which led in some cases to impulsive and harmful behaviour. Processes associated with rash reactivity and inadequate deliberation were recognised as proximal and dynamic risk factors for self-harm for some. Increased premeditation, potentially as an artefact of age and maturity, conferred a protective influence. Findings are in line with theoretical path models of impulsivity that combine emotional, cognitive, motivational, and behavioural domains in predicting psychopathology. The card-sort methodology helped to facilitate frank discussion. Young people were able to describe their behaviours and to articulate the cognitive processes beneath them.

## Supporting information

S1 File Topic guide and interview schedule. (DOCX)
