# Supplementary material for: What young people say about impulsivity in the short-term build up to self-harm: A qualitative study using card-sort tasks
Source: PLoS One. 2020 Dec 21;15(12):e0244319. doi: 10.1371/journal.pone.0244319 (PMC7751959; doi:10.1371/journal.pone.0244319)
Supplement: S1 File — (DOCX) [file pone.0244319.s001.docx]

**Topic guide and interview schedule: “What young people say about impulsivity in the short-term build up to self-harm. A qualitative study using card-sort tasks.**

*AIM: to talk with young people about their experiences of impulsivity and self-harm, to explore card selections during the card-sort tasks. To use open-ended questions, follow up questions and prompts. Welfare checks ongoing throughout session. Close with safeguarding.*

Suggested preamble and prompts:

“You’ve talked to me a little about how you see yourself, and your experience and thoughts and feelings in the build-up to self-harm in the card sort tasks. Now I’d like to explore some of the things you’ve identified in a little more detail and to talk to you about some of the other times you might have self-harmed (or thought about it) and how you might have been thinking and feeling then. Is this ok?”

Background:

Q1. To begin, can you tell me a little more about your history with self-harm? *[Prompts - for example, How old were you when you first thought about self-harm and first self-harmed? When was the last time you self-harmed? How frequently might you self-harm?* Can you tell me a little about the methods that you might use to self-harm?*]*

Q2. Can you tell me about any support you are currently receiving for self-harm? Have you talked to anyone about your self-harm before? Is this something you think you could do? *[Explore feelings around support/signposting…pick up at end of interview]*

Q3. Looking at the selections you’ve made for the card-activity both in terms of the cards about you, and the ones about your recent experiences of self-harm… *[card selection remains visible]* I’d like to talk about some of your choices. How did you find that experience?  Did you identify items that you had not thought about before? Or were surprising? Please explain…Etc.

*(Drawing on the card selections relating to impulsivity).*

Q4. We’ve talked a bit in the survey and in the card activity about impulsivity and that there are different ways we can understand impulsivity. One way is that we might act a bit rashly when we are feeling strong emotions. *[Check if selected these cards for card-activity].* Can you tell me a little more about if and how this way of responding to emotion seems relevant in your experience…Have you any other thoughts about this and how it might relate to you and the way you behave in other instances?

Q5. Another way of thinking about impulsiveness is as a way of responding in which we tend to make quick decisions without thinking through the consequences, or worrying about what those consequences might be? So impulsivity might be less about reacting to emotions, and more about how we think and process things? Does this seem relevant in your experience? – Can you tell me more etc?

Are there other ways of being impulsive that feel relevant to you? *[Explore other conceptions drawing on card choices where appropriate.]*

Q6. Thinking about all these things we’ve been talking about, I’d like to ask you a little about the *first* time you self-harmed. Can you recall that experience? If you can, please explain a little how you might have been thinking and feeling at that time?

Q7. Looking at the cards you’ve selected for your most recent episode of self-harm experience *[Card selection remains visible],* do you think you would have picked different cards or removed cards if you were describing that first experience? Please explain. How do you think impulsivity *(e.g. relating to emotion, not thinking through consequences, acting rashly when bored, or needing stimulation)* might have been relevant to you that first time? Please explain to me if you can. Do you have different expectations of self-harm now than you did then? Please explain. How have your motivations changed, if at all?

Q8. Are there times in which you’ve thought about self-harm, but not gone on to self-harm? Can you talk to me about the reasons why this might be? *(Explore if relevant, self-control, emotional awareness, resisting urges.)*

Q9. Do you think some of the things we’ve spoken about *[like responding to emotion, thinking about the consequences of our actions, acting differently when we are bored, or want stimulation, being able to resist our urges etc]* might make a difference to you, in stopping you self-harm, or continuing to self-harm? In what ways?

Q10. I’ve asked you a lot of questions! I wondered if there was anything you’d like to ask me about what we have been talking about?

*Ending and Welfare check ”How have you found taking part? How are you feeling now?*

*Visual analogue mood scale 1-10. Discuss. Debrief. Signposting.*
